# Out-of-hospital cardiac arrest in residential aged care facilities is independently associated with lower survival in Perth, Australia

**DOI:** 10.1016/j.resplu.2023.100495

**Published:** 2023-11-16

**Authors:** Milena Talikowska, Stephen Ball, David Majewski, Jason Belcher, Rudolph Brits, Sheryl Gallant, Lyndall Finn, Judith Finn

**Affiliations:** a Prehospital, Resuscitation and Emergency Care Research Unit (PRECRU), Curtin School of Nursing, Curtin University, Bentley, Western Australia, Australia; b St John WA, Belmont, Western Australia, Australia; c Medical School (Emergency Medicine), The University of Western Australia, Nedlands, Western Australia, Australia; d School of Public Health and Preventive Medicine, Monash University, Melbourne, Victoria, Australia

**Keywords:** out-of-hospital cardiac arrest, resuscitation, survival, residential aged care, nursing home

## Abstract

**Aim:**

To compare out-of-hospital cardiac arrest (OHCA) characteristics and outcomes between people aged ≥ 65 years who arrested in a residential aged care facility (RACF) versus a private residence in Perth, Australia.

**Methods:**

We undertook a retrospective cohort study of OHCA cases attended by emergency medical services (EMS) in Perth, January 2018-December 2021. OHCA patient and event characteristics and survival outcomes were compared via univariate analysis. Multivariable logistic regression was used to investigate the relationship between residency type and (i) return of spontaneous circulation (ROSC) at emergency department (ED) and (ii) 30-day survival.

**Results:**

A total of 435 OHCA occurred in RACFs versus 3,395 in private residences. RACF patients were significantly older (median age: 86 [IQR 79, 91] vs 78 [71, 85] years; *p* < 0.001), more commonly female (50.1% vs 36.8%; *p* < 0.001), bystander-witnessed arrests (34.9% vs 21.5%; *p* < 0.001), received bystander cardiopulmonary resuscitation (42.1% vs 28.6%; *p* < 0.001), had less shockable first monitored rhythms (4.0% vs 8.1%; *p* = 0.002) and more frequently had a “do not resuscitate” order identified (46.0% vs 13.6%; <0.001). Among those with EMS-attempted resuscitation or with defibrillation before EMS arrival, ROSC at ED and 30-day survival were significantly lower in the RACF group (6.2% vs 18.9%; *p* < 0.001 and 1.9% vs 7.7%; *p* < 0.001). The adjusted odds of ROSC at ED (aOR: 0.22 [95%CI: 0.10, 0.46]) and 30-day survival (aOR: 0.20 [95%CI 0.05, 0.92]) were significantly lower for RACF residents.

**Conclusion:**

RACF residency was an independent predictor of lower survival from OHCA, highlighting the importance of end-of-life planning for RACF residents.

## Introduction

With an aging population in Australia, an increasing number of people are predicted to be living in residential aged care facilities (RACF) in the future.^1^ Projections suggest the number of people aged ≥85 years will double to quadruple over the next half-century, from 555,000 in 2022^2^ to between 1.35 and 2.16 million by 2066.^3^, ^4^ It has been shown that older people living in RACFs experience higher rates of emergency medical service (EMS) attendance, up to four times those for age- and sex-matched people living in the community.^5^ Given the increased risk of out-of-hospital cardiac arrest (OHCA) in adults over the age of 65 years,^6^, ^7^ one might expect a number of these EMS calls to RACFs to relate to OHCA. However, survival from OHCA in RACF patients is commonly reported to be lower than for OHCA among older people living in private residences.^8^, ^9^, ^10^, ^11^, ^12^ For example, in Melbourne (Australia), Deasy et al. showed that survival from OHCA among RACF residents was significantly lower than for people aged 70 years or older who arrested ‘at home’ (1.8% vs 4.7%; *p* = 0.001).^8^ Paratz et al. in the Australian state of Victoria likewise demonstrated that the odds of surviving to discharge were significantly lower for people aged ≥80 years who arrested in a ‘nursing home’ compared to ‘at home’ (OR: 0.47 [95%CI 0.34, 0.64]).^13^ In another Australian study, survivors in RACFs were reported to have poor 12-month functional recovery.^14^ International studies from Denmark^9^, Japan,^15^ Hong Kong^10^, Ontario^11^ and Singapore^12^ also showed poorer survival outcomes for people who arrested in a RACF or ‘nursing home’ compared to those who arrested in the community. However, a French study by Vaux et al. reported that although prehospital return of spontaneous circulation (ROSC) was lower among those who arrested in a nursing home versus their own home (14% vs 16%; *p* = 0.03), the difference in 30-day survival was not significant between the two groups (2.0% vs 1.8%; *p* > 0.05).^16^ Shibahashi et al. in Tokyo found a significantly higher 30-day survival among nursing home residents versus those who arrested in a private residence (2.6% vs 1.8%; *p* < 0.001)^17^; attributed to increased likelihood of the arrest being witnessed and higher rates of bystander cardiopulmonary resuscitation (CPR) and shock delivery using an automated external defibrillator (AED). Kitamura et al. in Osaka reported that the adjusted odds of neurologically favourable outcome among elderly patients with bystander-witnessed OHCA of cardiac aetiology did not differ significantly between arrests that occurred in a nursing home versus in a private home (aOR: 0.81 [95%CI 0.47, 1.42]).^18^ Given the variability in reported survival outcomes, we sought to investigate OHCA in RACF patients in a population-based cohort in Perth, Australia. Specifically, we aimed to describe OHCA event and patient characteristics and survival outcomes in people aged ≥65 years who arrested in a RACF versus in a private residence.

## Methods

### Design

We undertook a retrospective cohort study of OHCA cases attended by St John Western Australia (SJWA) EMS paramedics in metropolitan Perth between 1 January 2018 and 31 December 2021 (4 years), for patients aged ≥65 years. We compared patients who arrested in a RACF to those who arrested in a private residence. RACF residents were identified by incident address and/or paramedics’ description of location in the electronic patient care record (ePCR). We excluded people who arrested in a RACF but were not residents (identified from patient residential address and/or ePCR paramedic description). We also excluded people who arrested in other types of facilities providing nursing services that were not specifically for the aged, for example, disability group homes. If patients arrested within an independent living unit located in a retirement village, we classified them in the ‘private residence’ category. If a patient arrested on a site that contained both independent living units and a RACF and it was not clear in which section the patient resided, we excluded that case from analysis. While there are no specific age limitations for entering a RACF in Australia, they are targeted towards people aged 65 years and older.^19^ Therefore we restricted our analysis in both the RACF and ‘private residence’ categories to people aged ≥65 years.

### Setting

The state of Western Australia (W.A.) has a population of 2.7 million,^20^ the majority of whom reside in the capital, Perth (2.1 million).^21^ In 2022 in Perth there were 14,232 people in permanent residential care and 241 using respite care.^22^ SJWA is the sole provider of road emergency ambulance services in Perth.^23^ During the study period, SJWA attended approximately 2,000 OHCA cases per year in metropolitan Perth; 3–4% of which occurred in a RACF.^23^, ^24^, ^25^, ^26^ Within metropolitan Perth, OHCA elicits a double vehicle (or where available, triple) Priority 1 ‘lights and sirens’ response by SJWA, with the exception of ‘obvious or expected deaths’, which receive a single vehicle Priority 1 response.^27^

### Data source

Our data were sourced from the SJWA OHCA Database maintained by the Prehospital, Resuscitation and Emergency Care Research Unit (PRECRU) at Curtin University. Patient and event data were captured from ePCRs, populated by attending paramedics and supplemented with computer-aided dispatch (CAD) data; 30-day survival was ascertained from the W.A. Death registry (or W.A. Cemetery Records) for patients not identified as deceased on the ePCR.^7^, ^24^

### Statistical analysis

Univariate analyses compared OHCA patient and event characteristics by arrest location (RACF vs private residence). To test the observed differences for statistical significance we used a Mann-Whitney U Test for continuous, non-parametric data and a Chi-squared test for categorical data, with α = 0.05. We then compared patient and event characteristics by arrest location for the subset of patients who received EMS-attempted resuscitation. We compared two survival outcomes: (i) ROSC at ED and (ii) 30-day survival. We constructed a multivariable logistic regression model to investigate the relationship between ROSC at ED and RACF residential status, adjusting for Utstein factors^28^ known to influence survival (age, sex, first monitored rhythm, aetiology, witnessed status, bystander CPR, bystander AED use and EMS response time (from the time of receiving the emergency call to the time of EMS arrival on scene)). We repeated this for 30-day survival. All analyses were carried out in IBM SPSS version 29 (IBM, Armonk, NY).

### Ethics

Ethics approval for this study was provided by the Human Research Ethics Committee at Curtin University as a sub-study of the Western Australian Pre-hospital Care Record Linkage Project (HR128/2013); with approval from the SJWA Research Governance Committee.

## Results

**Fig. 1:**
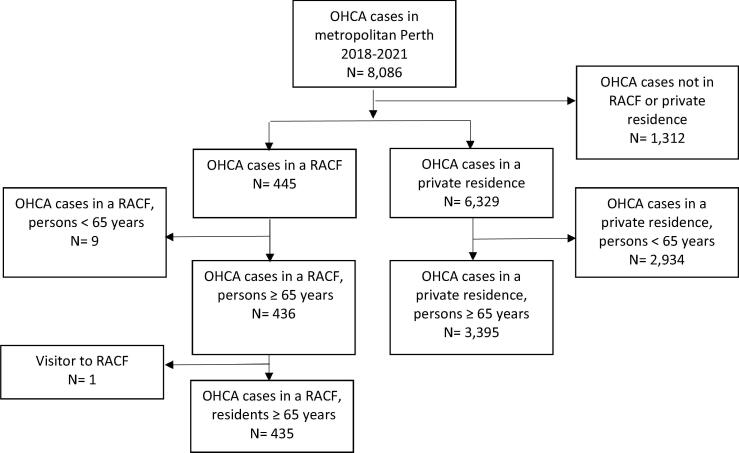
Flowchart for the selection of the study cohort.

Across all ages, there were 8086 OHCAs attended by SJWA in metropolitan Perth during the study period (1 January 2018 to 31 December 2021). Among the 4096 OHCA in patients aged ≥65 years, 435 (10.4%) occurred in a RACF while 3395 (81.1%) occurred in a private residence (only 266 [6.5%] occurred in a public location). We excluded one case where a visitor arrested in a RACF (Fig. 1).

Patients who arrested in a RACF were significantly older (median age 86 [interquartile range (IQR) 79, 91] vs 78 [71, 85] years; *p* < 0.001); more commonly female (50.1% vs 36.8%; *p* < 0.001), bystander-witnessed (34.9% vs 21.5%; *p* < 0.001) or EMS-witnessed arrests (15.2% vs 8.3%; *p* < 0.001), received bystander CPR (42.1% vs 28.6%; *p* < 0.001) and had a “do not resuscitate” order identified (46.0% vs 13.6%; <0.001). They were also less likely to have a shockable first monitored rhythm (4.0% vs 8.1%; *p* = 0.002) (Table 1).

**Table 1:** Patient and event characteristics for OHCA that occurred in a RACF vs. a private residence in patients aged ≥ 65 years in metropolitan Perth, 1 January 2018–31 December 2021.

**Characteristic**	**RACF (N = 435)**	**Private residence (N = 3395)**	**p-value**
Age (median (IQR)) years	86 (80, 91)	78 (71, 85)	<0.001
Female (%)	218 (50.1%)	1248 (36.8%)	<0.001
Witnessed arrest (%)			
*EMS-witnessed*	66 (15.2%)	281 (8.3%)	<0.001
*Bystander-witnessed*	152 (34.9%)	729 (21.5%)	<0.001
*Unwitnessed*	217 (49.9%)	2385 (70.3%)	<0.001
Bystander CPR (%)	183 (42.1%)	971 (28.6%)	<0.001
Bystander shock given (%)	0 (0.0%)	5 (0.1%)	0.423
EMS-attempted resuscitation (%)	162 (37.2%)	1317 (38.8%)	0.532
“Do not resuscitate” order^1^	200 (46.0%)	463 (13.6%)	<0.001
Shockable first monitored rhythm (%)	17 (4.0%)	273 (8.1%)	0.002
Presumed cardiac aetiology (%)	377 (86.7%)	3010 (88.7%)	0.221
Response time (median (IQR)) mins^2^	8.89 (6.82, 11.8)	8.78 (6.67, 11.5)	0.392

CPR: cardiopulmonary resuscitation; EMS: emergency medical services; IQR: interquartile range; OHCA: out-of-hospital cardiac arrest; RACF: residential aged care facility.

Notes:

1. This includes all patients who had a “do not resuscitate” (DNR) order, including those who received EMS-attempted resuscitation prior to the DNR being confirmed.

2. N = 5 cases were missing response time data.

There was no significant difference in the proportion of EMS-attempted resuscitation between the groups (37.2% in the RACF group vs 38.8% in the private residence group; *p* = 0.532). The above-mentioned differences in patient and event characteristics between the two groups remained statistically significant for the subset of patients who had EMS-attempted resuscitation (or bystander AED shock) (Table 2), except for the proportions of bystander-witnessed arrests and EMS-witnessed arrests, which ceased to be significantly different. In addition, the proportion of arrests of presumed cardiac cause was significantly lower in the RACF group versus the private residence group, among those who had an EMS resuscitation attempt or a bystander AED shock (87.7% vs 92.5%, *p* = 0.033). All crude survival outcomes were significantly lower in the RACF group (ROSC at ED: 6.2% vs 18.9%; *p* < 0.001 and 30-day survival: 1.9% vs 7.7%; *p* < 0.001).

**Table 2:** Patient and event characteristics and survival outcomes for OHCA that occurred in a RACF vs. a private residence in patients aged ≥ 65 years in metropolitan Perth, where resuscitation was attempted by EMS or an AED shock was delivered prior to EMS arrival, 1 January 2018–31 December 2021.

**Characteristic**	**RACF (N = 162)**	**Private residence (N = 1318)**	**p-value**
Age (median (IQR)) years	85 (78, 90)	77 (71, 84)	<0.001
Female (%)	84 (51.9%)	449 (34.1%)	<0.001
Witnessed arrest (%)			
*EMS-witnessed*	30 (18.5%)	249 (18.9%)	0.909
*Bystander-witnessed*	68 (42.0%)	555 (42.1%)	0.974
*Unwitnessed*	64 (39.5%)	514 (39.0%)	0.901
Bystander CPR (%)	114 (70.4%)	757 (57.4%)	0.002
Bystander shock given (%)	0 (0.0%)	5 (0.4%)	0.432
Shockable first monitored rhythm (%)	11 (6.9%)	269 (20.7%)	<0.001
Presumed cardiac aetiology (%)	142 (87.7%)	1219 (92.5%)	0.033
Response time (median (IQR)) mins^1^	9.00 (6.58, 12.0)	9.25 (7.03, 11.9)	0.423
“Do not resuscitate” order^2^	41 (25.3%)	154 (11.7%)	<0.001
ROSC at ED (%)	10 (6.2%)	249 (18.9%)	<0.001
Survived to 30 days (%)	3 (1.9%)	101 (7.7%)	0.006

AED: automated external defibrillator; CPR: cardiopulmonary resuscitation; ED: emergency department; EMS: emergency medical services; IQR: interquartile range; OHCA: out-of-hospital cardiac arrest; RACF: residential aged care facility; ROSC: return of spontaneous circulation.

Notes:

1. N = 1 case was missing response time data.

2. Patients who received EMS-CPR prior to “do not resuscitate” (DNR order being confirmed.

The adjusted odds of ROSC at ED (aOR: 0.22 [95%CI 0.10, 0.46]) (Table 3) and 30-day survival (aOR: 0.20 [95%CI 0.05, 0.92]) (Table 4) were significantly lower for RACF residents compared to people who arrested in a private residence.

**Table 3:** Multivariable logistic regression analysis for ROSC at ED.

Covariate	aOR (95% CI)	p-value
Nursing home resident	0.22 (0.10, 0.46)	<0.001
Age (years)	0.97 (0.94, 0.99)	<0.001
Male	0.67 (0.48, 0.92)	0.014
Shockable first monitored rhythm	5.75 (4.04, 8.17)	<0.001
Presumed cardiac aetiology	2.23 (1.13, 4.40)	0.021
Bystander-witnessed status		
*EMS-witnessed*	Reference	
*Bystander-witnessed*	0.16 (0.10, 0.25)	<0.001
*Unwitnessed*	0.04 (0.02, 0.06)	<0.001
Bystander shock given	2.73 (0.35, 21.3)	0.340
Bystander CPR	2.55 (1.72, 3.77)	<0.001
Response time (mins)	1.01 (0.99, 1.02)	0.504

aOR: adjusted odds ratio; CI: confidence interval; CPR: cardiopulmonary resuscitation; ED: Emergency department; EMS: Emergency medical services; ROSC: Return of spontaneous circulation

**Table 4:** Multivariable logistic regression analysis for 30-day survival.

Covariate	aOR (95% CI)	p-value
Nursing home resident	0.20 (0.05, 0.92)	0.038
Age (years)	0.94 (0.90, 0.97)	<0.001
Male	0.84 (0.48, 1.47)	0.538
Shockable first monitored rhythm	14.8 (8.41, 26.2)	<0.001
Presumed cardiac aetiology	6.57 (0.85, 51.0)	0.072
Bystander-witnessed status		
*EMS-witnessed*	Reference	
*Bystander-witnessed*	0.08 (0.04, 0.16)	<0.001
*Unwitnessed*	0.01 (0.003, 0.03)	<0.001
Bystander shock given	5.23 (0.41, 66.7)	0.202
Bystander CPR	1.26 (0.60, 2.66)	0.540
Response time (mins)	0.98 (0.95, 1.01)	0.260

aOR: adjusted odds ratio; CI: confidence interval; CPR: cardiopulmonary resuscitation; EMS: Emergency medical services.

## Discussion

We compared OHCA event and patient characteristics and survival outcomes for people aged ≥65 years who arrested in a RACF versus a private residence in Perth. Given the variability in reported survival outcomes in the literature, we present new findings that strengthen the existing evidence for lower survival from OHCA among RACF residents.^15^, ^9^, ^10^, ^11^, ^12^ We found that both ROSC at ED and 30-day survival were significantly lower in the RACF group compared to the private residence group (ROSC at ED: 6.2% vs 18.9% and 30-day survival: 1.9% vs 7.7%). These results are consistent with RACF residents tending to be older and presumably with more co-morbidities and/or greater frailty, necessitating their residence in the RACF. Even after adjustment for known prognostic (Utstein) factors, RACF residency was found to be an independent predictor of both lower odds of ROSC at ED (aOR: 0.22) and 30-day survival (aOR: 0.20). While the majority of evidence in the literature points towards lower survival after OHCA among RACF residents compared to those who arrested outside of the RACF, a systematic review and meta-analysis on this topic would quantify the effect.

Our univariate analyses present results that are consistent with those reported by others.^9^, ^10^, ^12^, ^15^, ^16^, ^18^, ^29^ RACF OHCA patients were significantly older (median age: 86 vs 78 years) and more commonly female (50% vs 37%). This finding makes sense given that eligibility for RACF admission is based upon the inability to live independently, which is more likely with older age. Furthermore, in Australia, like in many other jurisdictions, the average life expectancy for women is higher than men.^30^ Like other authors,^9^, ^16^, ^17^, ^29^ we found that OHCA in a RACF was significantly more likely to be witnessed, either by bystanders (35% in RACF vs 22% in a private residence) or by EMS (15% vs 8%). A finding of this nature could reasonably be expected given that RACFs are staffed 24/7. Furthermore, if staff notice a patient deteriorating, they may be more likely to call an ambulance prior to an arrest occurring, leading to more EMS-witnessed arrests. Similarly to others,^12^, ^15^, ^16^, ^9^, ^10^ we found that the proportion of bystander CPR was significantly higher for the RACF group compared to those who arrested in a private residence (42% vs 29%); this coincides with the higher proportion of witnessed arrests and the assumption that a number of RACF staff are trained in basic life support (BLS).

Among patients who received EMS-attempted resuscitation, 70% in the RACF group and 57% in the private residence group had bystander CPR. Of note, 30% in the RACF group did not receive bystander CPR. It is unclear why an emergency ambulance was called if residents were not provided with CPR by the RACF staff prior to the arrival of the paramedics. Studies show that numerous RACF lack a clear CPR policy.^31^, ^32^ In addition, Lee et al. reported that non-medical staff in a nursing home were less likely to initiate CPR than medical staff (doctors or nurses).^33^ In our study, while 4% of patients in the RACF group had a shockable first monitored rhythm recorded by SJWA paramedics, none of them received an AED shock prior to EMS arrival. Again, the reason for this is unclear; but it is possibly due to the lack of a clear resuscitation policy, lack of available AEDs in the RACF and/or lack of up-to-date training.

In metropolitan Perth, OHCA calls (with the exception of ‘expected’ or ‘obvious’ deaths)^27^ are dispatched with two ambulances and, where available, a third vehicle equipped with a mechanical CPR device. All vehicles respond with Priority 1 ‘lights and sirens’.^23^ However, ‘lights and sirens’ responses have been previously linked to an increased risk of road traffic accidents.^34^ In addition, dispatch of multiple vehicles to the RACF patient may limit the number of vehicles available to respond to other critical incidents in the community. One could argue that if an emergency response is requested for cardiac arrest in RACFs, then bystander interventions by RACF staff should be optimised prior to EMS arrival, including good quality CPR and AED application, in order to maximise the likelihood of patient survival.

Almost half of OHCA patients in the RACF were ultimately found to have a DNR order in place (46% vs 14% among those who arrested at home). Other studies report DNR order rates of 40%^35^ and 61%^36^ in the RACF setting. Among those in our study who received EMS-attempted resuscitation in a RACF, a quarter (25%) were ultimately found to have a DNR. This finding suggests that there may have been a delay between the arrival of the EMS on scene and the communication of the DNR to paramedics. Timely communication of a patient’s DNR status is critical to preventing unwanted resuscitation. If a patient’s DNR status could be easily identified and made known to the call-taker at the time of the emergency call, this would allow for appropriate resources, proportional to the patient’s requirements, to be dispatched from the start.

Our findings add to the body of evidence demonstrating that survival from OHCA is lower in the RACF setting. They also highlight the importance of end-of-life planning for RACF residents, giving due consideration of the realistic likelihood of survival.^37^ These findings provide an opportunity for EMS and RACF providers to engage to establish a joint approach to cardiac arrest events.

### Limitations

Our study has several limitations. Due to its observational nature, there is always an inherent risk of bias. In particular, there may be important differences in comorbidity between OHCA patients in RACFs and private residences, which were beyond the scope of our study to measure. In addition, SJWA has different guidelines for withholding resuscitation in RACF patients versus those who arrest in the community. Specifically, resuscitation can be withheld for RACF patients if they are aged ≥80 years and ‘obviously frail’ (corresponding to a Clinical Frailty Scale^38^ of 7, 8 or 9). For patients who arrest in the community, guidelines for withholding resuscitation have an additional requirement for asystole as the first monitored rhythm.^39^ This may represent an additional source of bias, however our analysis showed that asystole was the predominant initial presenting rhythm in the RACF group, with only 4% of patients having a shockable initial rhythm. Part of our cohort experienced an OHCA during the COVID-19 pandemic, however we have previously shown that COVID-19 had no apparent effect on OHCA incidence and outcome in Western Australia.^40^ Finally, some of our findings may not be applicable to other jurisdictions that have a different model of response to OHCA in RACFs.

## Conclusion

RACF residency was an independent predictor of lower survival from OHCA in people aged 65 years or over. Furthermore, almost half of EMS-attended OHCA patients in a RACF were ultimately found to have a DNR order in place. These findings highlight the importance of end-of-life planning and more timely identification of DNR status for RACF residents.

## Funding

Judith Finn is the recipient of a National Health and Medical Research Council (NHMRC) Investigator Grant #1174838. The Prehospital, Resuscitation and Emergency Care Research Unit (PRECRU) receives research funding from St John Western Australia (SJWA).

## CRediT authorship contribution statement

**Milena Talikowska:** Conceptualization, Data curation, Formal analysis, Methodology, Validation, Writing – original draft, Writing – review & editing. **Stephen Ball:** Conceptualization, Data curation, Formal analysis, Methodology, Validation, Writing – review & editing. **David Majewski:** Data curation, Writing – review & editing. **Jason Belcher:** Conceptualization, Methodology, Validation, Resources, Writing – review & editing. **Rudolph Brits:** Conceptualization, Resources, Writing – review & editing. **Sheryl Gallant:** Data curation, Investigation, Writing – review & editing. **Lyndall Finn:** Data curation, Investigation, Writing – review & editing. **Judith Finn:** Conceptualization, Formal analysis, Funding acquisition, Methodology, Supervision, Validation, Writing – review & editing.

## Declaration of competing interest

The authors declare the following financial interests/personal relationships which may be considered as potential competing interests: Jason Belcher and Rudolph Brits are employees of SJWA. Judith Finn and Stephen Ball hold adjunct research positions with SJWA. Milena Talikowska, David Majewski, Lyndall Finn Sheryl Gallant, Judith Finn and Stephen Ball are employees of the Prehospital, Resuscitation and Emergency Care Research Unit (PRECRU) at Curtin University; PRECRU receives research funding from SJWA. There are no other conflicts of interest to declare.
